# Supplementary material for: Co-Operative Biofilm Interactions between Aspergillus fumigatus and Pseudomonas aeruginosa through Secreted Galactosaminogalactan Exopolysaccharide
Source: J Fungi (Basel). 2022 Mar 24;8(4):336. doi: 10.3390/jof8040336 (PMC9030451; doi:10.3390/jof8040336)
Supplement: Supplementary file 1 [file jof-08-00336-s001.zip › jof-1624713-supplementary.pdf]

Supplementary Materials: The following are available online at https://www.mdpi.com/article/10.3390/jof8040336/s1, Figure S1: Dose-dependent deacetylation of GAG by recombinant Agd3, Figure S2: Recombinant Agd3 does not exhibit activity on *P. aeruginosa* biofilms, Table S1: A list of fungal and bacterial strains used in this study.

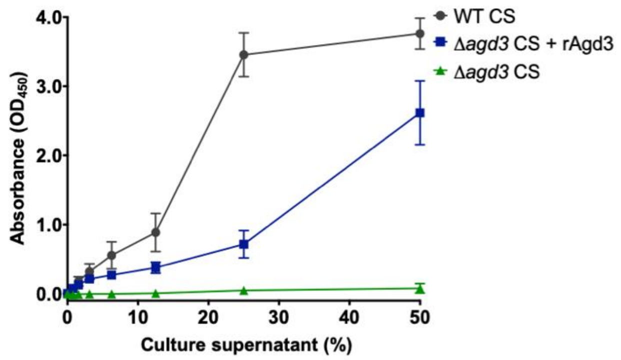

Supplementary Figure S1. Dose-dependent deacetylation of GAG by recombinant Agd3. Deacetylation of *N*-acetylated-GAG-containing *A. fumigatus* culture supernatants (Δ*agd3* CS) by 130 nM rAgd3 (rAgd3) or wild-type *A. fumigatus* culture supernatants (WT CS) was determined with GAG-enzyme-linked lectin assay (GAG-ELLA). Dots represent the means ± standard deviations of 4 independent experiments.

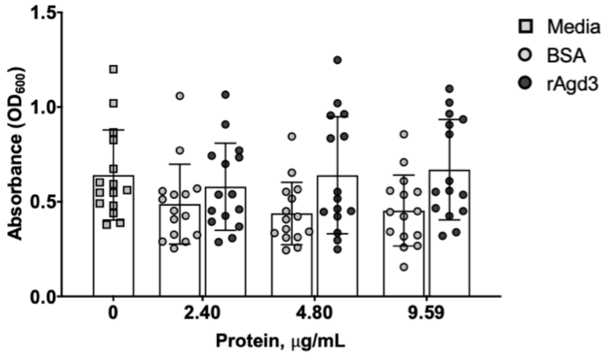

Supplementary Figure S2. Recombinant Agd3 does not exhibit activity on *P. aeruginosa* biofilms. Biofilm formation by wild-type *P. aeruginosa* PA14 grown in the presence of either bovine serum albumin (BSA) or recombinant Agd3 (rAgd3) was quantified with crystal violet staining. Bars represent the means ± standard deviations of 3 independent experiments. Note there is no significant difference between BSA and rAgd3 treated samples ($P > 0.3431$) as determined by paired t test.

Supplementary Table S1. The list of fungal and bacterial strains used in this study.

Strain or genotype	Referred to in figures (referred to in text)	Description	Source
**Plasmids**			
pMKB1::*mCherry*	pMKB1::*mCherry*	Constitutive mCherry expression and carbenicillin resistance	Sam Moskowitz
pMKB1::*gpf*	pMKB1::*gpf*	Constitutive GFP expression and carbenicillin resistance	Sam Moskowitz
pGFP-*ble*	pGFP-*ble*	*egfp* (GFP) expression and *ble* (phleomycin) resistance under constitutive expression of *gpdA* promoter via osmotic signals	[71]
Aspergillus fumigatus			
Af293	WT *A. fumigatus* (wild-type *A. fumigatus*)	*A. fumigatus* wild type strain	Paul T. Magee
Af293 Δ*uge3*	Δ*uge3* (GAG-deficient *A. fumigatus*)	Non-polar *uge3* open reading frame deletion	[23]
Af293 Δ*agd3*	Δ*agd3* (Agd3-deficient *A. fumigatus*)	Non-polar a*gd3* open reading frame deletion	[24]
Af293 AF-eGFP	WT *A. fumigatus* (GFP-producing *A. fumigatus*)	Chromosomal insertion of plasmid pGFP-*ble* for GFP expression	[71]
Af293 Δ*uge3*-eGFP	Δ*uge3* (GFP-producing *A. fumigatus*)	Chromosomal insertion of plasmid pGFP-*ble* for GFP expression	This study
Pseudomonas aeruginosa			
PA14	PA14 (wild-type *P. aeruginosa*)	*P. aeruginosa* wild-type strain	Deborah A. Hogan
PA14-p*mCherry*	PA14 (mCherry-producing *P. aeruginosa*)	Constitutive mCherry expression	This study
PA14 Δ*pelA*	Δ*pelA* (Pel-deficient *P. aeruginosa*)	Non-polar *pelA* allelic replacement	Deborah A. Hogan
PA14 Δ*pelA*-p*mCherry*	Δ*pelA* (mCherry-producing *P. aeruginosa*)	Constitutive mCherry expression	This study
PAO1 Δ*wspF*Δ*psl*P_{BAD}*pel*	$P_{BAD}$$pel^{+}$ (Pel-overproducing *P. aeruginosa*)	Non-polar *wspF* deletion; polar *pslBCD* of *psl* operon deletion; *pel-araC*-P_{BAD} promoter replacement for L-arabinose-inducible *pel* operon	[28]
PAO1 Δ*wspF*Δ*psl*P_{BAD}*pel* pRFP	$P_{BAD}$$pel^{+}$ (Pel-overproducing mCherry *P. aeruginosa*)	Constitutive mCherry expression	[42]
PA14-p*gfp*	PA14 (GFP-producing *P. aeruginosa*)	Constitutive GFP expression	This study
PA14 Δ*pelA*-p*gfp*	Δ*pelA* (GFP-producing *P. aeruginosa*)	Constitutive GFP expression	This study

Abbreviations: GFP, green-fluorescent protein.
